# Selection of antisense oligonucleotides based on multiple predicted target mRNA structures

**DOI:** 10.1186/1471-2105-7-122

**Published:** 2006-03-09

**Authors:** Xiaochen Bo, Shaoke Lou, Daochun Sun, Wenjie Shu, Jing Yang, Shengqi Wang

**Affiliations:** 1 Beijing Institute of Radiation Medicine, 27 Taiping Road, Beijing 100850, P R China

## Abstract

**Background:**

Local structures of target mRNAs play a significant role in determining the efficacies of antisense oligonucleotides (ODNs), but some structure-based target site selection methods are limited by uncertainties in RNA secondary structure prediction. If all the predicted structures of a given mRNA within a certain energy limit could be used simultaneously, target site selection would obviously be improved in both reliability and efficiency. In this study, some key problems in ODN target selection on the basis of multiple predicted target mRNA structures are systematically discussed.

**Results:**

Two methods were considered for merging topologically different RNA structures into integrated representations. Several parameters were derived to characterize local target site structures. Statistical analysis on a dataset with 448 ODNs against 28 different mRNAs revealed 9 features quantitatively associated with efficacy. Features of structural consistency seemed to be more highly correlated with efficacy than indices of the proportion of bases in single-stranded or double-stranded regions. The local structures of the target site 5' and 3' termini were also shown to be important in target selection. Neural network efficacy predictors using these features, defined on integrated structures as inputs, performed well in "minus-one-gene" cross-validation experiments.

**Conclusion:**

Topologically different target mRNA structures can be merged into integrated representations and then used in computer-aided ODN design. The results of this paper imply that some features characterizing multiple predicted target site structures can be used to predict ODN efficacy.

## Background

Antisense oligonucleotides (ODNs) have served as powerful tools during the post-genome era. They provide an important approach to sequence-specific knockdown of gene expression, offering significant advantages over gene knockout techniques in respect of cost, time and resource requirements, and have therefore been widely used for determining gene function, validating drug targets and elucidating pathways [1,2]. ODNs also have potential as novel therapeutic agents for various diseases; several antisense compounds have been evaluated in clinical trials with promising results [3].

However, even with careful design, only a small proportion of ODNs against a given RNA effectively suppress the target gene in living cells [4]. It is commonly accepted that the identification of accessible sites in the target RNA is of great importance in designing ODNs. Various experimental approaches to the identification of promising local target sites have been described in recent years [5-10]. There has also been much interest in computational approaches to ODN design, which have advantages over experimental methods in terms of throughput, cost and efficiency. Several approaches to efficacy prediction have been proposed for rational selection of ODN target sites [11-14].

Among the factors that influence the activity of a given ODN, the local secondary structures of the target mRNA are very significant in determining *in vitro *efficiency [5,15-17] and are therefore particularly important in current ODN design strategies [18-20]. Local target site structures have also been used as the basis of rational design for other kinds of nucleic acids drugs such as antisense RNAs [21], catalytic RNAs [22] and ribozymes [23]. However, the term "structure" in these studies refers to "single computational predicted structure", not the real structure of the target mRNA; RNA secondary structure is difficult to determine experimentally.

Many RNA secondary structure prediction algorithms have been proposed during the past 20 years. Since the thermodynamically most stable structure of a molecule is generally the one with the minimum free energy (MFE), the initial aim of these prediction methods is to determine the MFE structure [24]. Several MFE structure searching algorithms have been described and are widely used in related research [25,26], especially in ODN target selection. However, partly because of the relatively low reliability of individual target mRNA structure predictions, researchers have often drawn inconsistent conclusions about favorable local structure motifs. The results obtained by Lima *et al*. [18] and Thierry *et al*. [19] indicated that single-stranded hairpin loops in RNA were the best target sites, whereas the studies by Laptev *et al*. [20] suggested that ODNs targeted to sequences predicted to form clustered double-stranded structures in RNA transcripts had the best potential.

It is also possible to consider conformations close to the energy minimum, and algorithms for calculating suboptimal structures within certain energy limits have been proposed [27,28]. The popular RNA secondary structure prediction program MFold now provides results over a range of free energies, mitigating the uncertainty of MFE prediction. Although multiple predicted structures are apparently more reliable, the MFE structure of the target mRNA is still used as the only structural basis in some ODN research. The main difficulty may lie in how to use these foldings simultaneously, since they can be topologically very different.

Studies on ensembles of target structures in ODNs design date back to Jaroszewski *et al*. [29], who considered the 30 lowest-energy computer-simulated structures of rabbit *β*-globin mRNA qualitatively. In some thermodynamic models, multiple predicted target structures have been merged into the form of free energy [30,31]. The earliest work on computational ODN design based on the original forms of multiple predicted target mRNA structures was perhaps that of Patzel *et al*. [17]. Five structures with low energy were predicted and aligned for a given sequence stretch, and ODN sequences were chosen if potentially favourable local structural elements occurred in all five. *In vitro *experiments showed that this theoretical protocol increased the statistical probability of identifying local target sites accessible to ODN sequences [17,32]. Another way to explore the original forms of optimal and suboptimal mRNA structures simultaneously, which is probably more straightforward, is to merge them into a single-stranded probability profile (SSPP), **P **= {*p*_*i*_}, 1 ≤ *i *≤ *n*, where *p*_*i *_is the probability that base *i *is single-stranded. Actually, algorithms for predicting single-stranded regions in RNA secondary structures have long been of interest, since such regions play many important roles in RNA-RNA, RNA -DNA and RNA-protein interactions [33]. The SFold web server [34] can now directly output the SSPP of an RNA molecule instead of definite individual structures. Ding and Lawrence [33] presented a method for predicting accessible sites in the SSPP of rabbit *β*-globin mRNA, obtained by summing statistical samples of probable secondary structures. Their results showed a significant correlation between the predicted hybridization potential and the degree of inhibition of *in vitro *translation. Some researchers regard this method as the most successful [11,12].

The original RNA structural information is used in essentially different ways in the two methods described above. In the method based on structure alignment, favorable structural elements are identified by base pairing patterns, which can be illustrated as graphs. The role of secondary structures in this method is similar to its role in earlier studies of ODN design based on the target mRNA MFE structure. The success of this method relies mainly on the greatly increased reliability of structural elements. However, in the method based on SSPP, the RNA structures resemble a special time series rather than molecular "structures" in the usual sense. Base pairing patterns, or topological features, can hardly be explored in SSPP. The common ground between these two methods is the emphasis on the role of single-stranded regions in determining target accessibility. In the SSPP of rabbit *β*-globin mRNA, Ding and Lawrence found a significant correlation between the peak value of SSPP and the degree of inhibition of translation. The "well-chacterized" single-stranded regions were revealed by high probability peaks in the profile [33], while in the systematic alignment of multiple predicted target mRNA secondary structures, large (>10 nt) consecutive sequence stretches not involved in base pairing were regarded as favorable structural motifs [17]. Since these two methods were only evaluated on a single target mRNA, further research is needed on a broad range of target genes.

The purpose of this article is to systematically explore the methods for computational selection of ODN target sites based on features defined in multiple predicted structures of the target mRNA. In our approach, the predicted mRNA structures were first merged into integrated representations. Efficacy-associated features were then screened from a set of features defined on these representations. The potential of neural networks for predicting efficacy on the basis of these features was also validated.

## Results

### Dataset

Three ODN databases have been reported: ODNBase [35], AOdb [12] and an unnamed database with experimental data from Isis Pharmaceuticals [36]. We have also developed a database named AOBase [37] (NAR molecular biology database collection entry number 781) for both the selection and design of ODNs. Currently, it stores 705 ODNs from the published literature tested against transcripts of 54 different target genes. Since no homogeneous database is publicly available, we perforce used a heterogeneous collection of measurements made by different researchers using different experimental techniques as our dataset. Four hundred and forty-eight ODNs against 28 different mRNAs were collected from AOBase to construct this dataset; 54.2% of them had been tested at protein level and the others at mRNA level. The data selection criteria were similar to those used in other ODN efficacy prediction studies [11-13]: (a) at least 4 ODNs were tested under the same experimental conditions; (b) ODN efficacies were presented as percentages of the control target gene expression level; (c) virus targets were excluded; (d) ODNs targeting to the translational initiation site were excluded, since regions surrounding the initiation codon are generally considered to be free of secondary structure [38]. To keep in line with most of the research on drug design, the ODN efficacies in our dataset were transformed into [100%-(% of control expression)].

RNA folding calculation times have been greatly reduced in recent years because of faster computers and improved algorithms. The MFold web server [39] can now fold 6000 bases for a batch job, which meets the need of full-length mRNA structure prediction in most cases and is therefore used in this study. Because the number of predicted suboptimal RNA secondary structures increases exponentially as the folding energy increases [40], only structures within 5 percent of the computed minimum free energy were taken into consideration. The upper bound on the number of simultaneously predicted structures was set to 50 to avoid the high computational cost of long RNA sequences. These settings were the default settings of the MFold web server. Table 1 is a brief summary of the dataset.

### Integrating multiple predicted target mRNA secondary structures

In this study, two methods were used to represent the multiple predicted local structures of target sites synthetically. All the predicted local structures were first merged into an SSPP, which is easily calculated from the ss-count file in the MFold output. For a more illustrative representation of the multiple predicted structures, the SSPP was further transformed to a "single-stranded/pair/uncertain" sequence (SUP representation) **S **= {*s*_*i*_}, where *s*_*i *_= '**S**' if base *i *is single-stranded, *s*_*i *_= '**P**' if base *i *is paired with another base, and *s*_*i *_= '**U**' if it is uncertain whether base *i *is single-stranded. The thresholds suggested by Ding and Lawrence [33] were used to map SSPP {*p*_*i*_} into the SUP representation {*s*_*i*_}, giving

Si={'S',pi>0.5'U',0.5≥pi>0.2'P',pi≤0.2     (1)
 MathType@MTEF@5@5@+=feaafiart1ev1aaatCvAUfKttLearuWrP9MDH5MBPbIqV92AaeXatLxBI9gBaebbnrfifHhDYfgasaacH8akY=wiFfYdH8Gipec8Eeeu0xXdbba9frFj0=OqFfea0dXdd9vqai=hGuQ8kuc9pgc9s8qqaq=dirpe0xb9q8qiLsFr0=vr0=vr0dc8meaabaqaciaacaGaaeqabaqabeGadaaakeaacqWGtbWudaWgaaWcbaGaemyAaKgabeaakiabg2da9maaceaabaqbaeGabmGaaaqaaiabcEcaNiabbofatjabcEcaNiabcYcaSaqaaiabdchaWnaaBaaaleaacqWGPbqAaeqaaOGaeyOpa4JaeGimaaJaeiOla4IaeGynaudabaGaei4jaCIaeeyvauLaei4jaCIaeiilaWcabaGaeGimaaJaeiOla4IaeGynauJaeyyzImRaemiCaa3aaSbaaSqaaiabdMgaPbqabaGccqGH+aGpcqaIWaamcqGGUaGlcqaIYaGmaeaacqGGNaWjcqqGqbaucqGGNaWjcqGGSaalaeaacqWGWbaCdaWgaaWcbaGaemyAaKgabeaakiabgsMiJkabicdaWiabc6caUiabikdaYaaaaiaawUhaaiaaxMaacaWLjaWaaeWaaeaacqaIXaqmaiaawIcacaGLPaaaaaa@5A1B@

**Figure 1:**
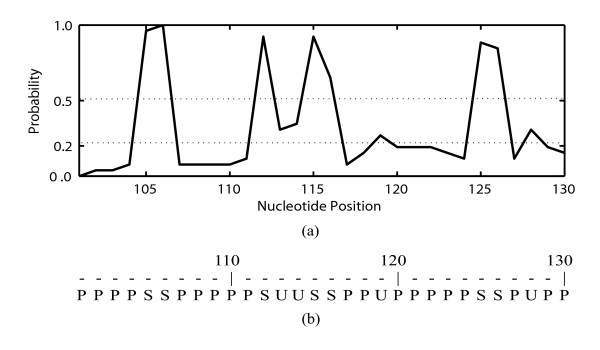
Two representations of multiple predicted structures of rabbit β-globin mRNA (G101-G130). (a) Single-stranded probability profile; (b) 'SUP' representation.

SUP representation loses a lot of structural information in comparison to graphical illustration or dot-parenthesis notation of RNA secondary structure and therefore cannot be used to explore the whole RNA structure. However, for RNA local structural analysis, especially of very RNA short regions, SUP gives a competent simplified representation. Figure 1 illustrates part of these two representations (101–130 nt) of rabbit *β*-globin mRNA structure.

### Selection of efficacy-associated features

The first important step in computational design based on multiple predicted mRNA structures is to find the efficacy-associated features in the SSPP and SUP representations of the target sites. Since the data structures of these two linear representations of multiple predicted structures are very different from graphical illustrations of RNA molecules, the topological features known to be correlated with efficacy must be redefined. However, new representations also afford opportunities to discover novel efficacy-associated features.

A set of features characterizing the local multiply-predicted target mRNA secondary structures was derived. Seven of these features were defined on the SSPP representation (listed in Table 2) while the other eleven were defined on the SUP sequence representation (listed in Table 3). The size of the local target, *n*, in the definition of features is equal to the length of the ODN.

The mean of all single stranded probabilities within a given target site, *f*_*mean*_, indicates the probability that the target site is single-stranded. The maximum value, *f*_*max*_, has also been used for this purpose [33]. *f*_*impulse*_, can be viewed as a relative peak value compared to the mean. The other statistics, *f*_*rms*_, *f*_*peak*_, *f*_*wave*_, and *f*_*difference*_, describe the structural consistency of the target site.

Numerical features defined on the SUP sequence are directly derived from research results and from empirical rules about target site selection based on local structure. Features *f*_*NS*_, *f*_*NP*_, *f*_*PS*_, and *f*_*PP*_, give an overall description of target structure, while *f*_5*S*_, *f*_5*P*_, *f*_3*S *_and *f*_3*P *_emphasize the local structure of the target site termini. Factors *f*_*CS *_and *f*_*CP *_are derived to confirm whether the occurrence of consecutive subsequences in single-stranded or helical regions is correlated with efficacy, as explored by Patzel *et al*. [17].

**Figure 2:**
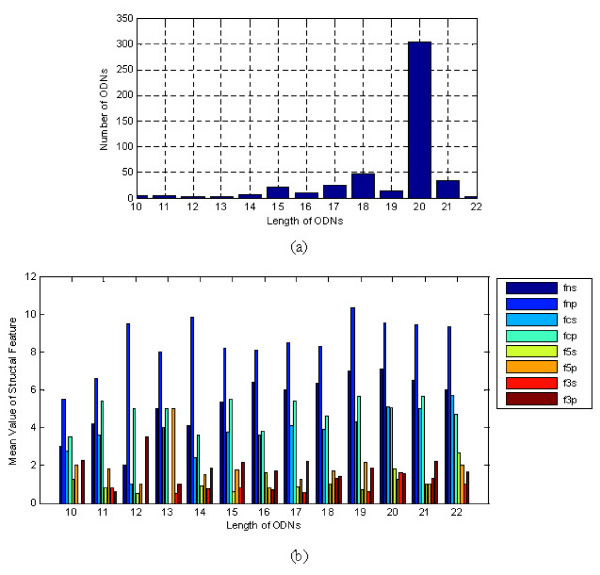
The distribution of ODN length and length-limited features. (a) The distribution of ODN lengths in the dataset; (b) Mean values of some features of ODNs with different lengths.

Absolute numbers of bases appear in the definitions of eight features defined on the SUP representation, viz. *f*_*NS*_, *f*_*NP*_, *f*_*CS*_, *f*_*CP*_, *f*_5*S*_, *f*_5*P*_, *f*_3*S *_and *f*_3*P*_. Since the ODN lengths in the dataset are not uniform, it is necessary to determine whether these features are bound up with or limited by the size of local target. Figure 2(a) shows the distribution of ODN lengths in the dataset, which range from 10 nt to 22 nt. Most of the ODNs were 20 nt long. The dataset was divided into groups according to ODN length. The mean values of these features were calculated for each group and are shown in Figure 2(b), which indicates no obvious relationships between these features and target size.

Two types of indices, efficiency prediction potential and classification potency, were used to measure the suitability of these parameters for rational ODN design. The efficacy prediction potential was evaluated by calculating the correlation between the features and efficacy, using Pearson linear correlation, Spearman rank correlation and Kendall rank correlation. The classification potency was evaluated by exploring the performance of Fisher linear discriminators, using the feature as the single independent variable. The performance was measured as specificity Sp=TnTn+Fp
 MathType@MTEF@5@5@+=feaafiart1ev1aaatCvAUfKttLearuWrP9MDH5MBPbIqV92AaeXatLxBI9gBaebbnrfifHhDYfgasaacH8akY=wiFfYdH8Gipec8Eeeu0xXdbba9frFj0=OqFfea0dXdd9vqai=hGuQ8kuc9pgc9s8qqaq=dirpe0xb9q8qiLsFr0=vr0=vr0dc8meaabaqaciaacaGaaeqabaqabeGadaaakeaacqWGtbWudaWgaaWcbaGaemiCaahabeaakiabg2da9maalaaabaGaemivaq1aaSbaaSqaaiabd6gaUbqabaaakeaacqWGubavdaWgaaWcbaGaemOBa4gabeaakiabgUcaRiabdAeagnaaBaaaleaacqWGWbaCaeqaaaaaaaa@39B4@ and sensitivity Se=TpTp+Fn
 MathType@MTEF@5@5@+=feaafiart1ev1aaatCvAUfKttLearuWrP9MDH5MBPbIqV92AaeXatLxBI9gBaebbnrfifHhDYfgasaacH8akY=wiFfYdH8Gipec8Eeeu0xXdbba9frFj0=OqFfea0dXdd9vqai=hGuQ8kuc9pgc9s8qqaq=dirpe0xb9q8qiLsFr0=vr0=vr0dc8meaabaqaciaacaGaaeqabaqabeGadaaakeaacqWGtbWudaWgaaWcbaGaemyzaugabeaakiabg2da9maalaaabaGaemivaq1aaSbaaSqaaiabdchaWbqabaaakeaacqWGubavdaWgaaWcbaGaemiCaahabeaakiabgUcaRiabdAeagnaaBaaaleaacqWGUbGBaeqaaaaaaaa@39A2@. Two different efficacy threshold values, 50% and 75%, were used to distinguish between positive and negative cases in our dataset, since these indices depend on threshold. Features matching at least one of the following two criteria were selected as efficacy-associated: (a) statistically significant correlation (*p *< 0.05) with efficacy; and (b) high specificity (≥0.7) or high sensitivity (≥0.7) in distinguishing between active and inactive ODNs.

The correlation between parameters and efficacy is presented in Table 4. Only four features defined on SSPP, i.e. *f*_*rms*_, *f*_*max*_, *f*_*peak *_and *f*_*difference*_, correlated strongly with efficacy. Table 5 compares the Fisher discrimination results for each parameter and different thresholds, indicating that *f*_*rms*_, *f*_*max*_, *f*_*peak*_, *f*_*difference*_, *f*_*PP*_, *f*_*CS*_, *f*_*CP*_, *f*_5*S *_and *f*_3*S *_can be used to distinguish between active and inactive ODNs according to our criteria.

The most noteworthy finding is that ODN efficacy seems not to rely greatly on the degree of single-strandedness in its target site, as suggested in previous publications [18-20], since *f*_*mean*_, *f*_*NS *_and *f*_*PS *_show neither sufficient correlation with efficacy nor good performance in identifying active ODNs. The lengths of consecutive single-stranded regions in the target site, which are characterized by *f*_*CS*_, prove useful for identifying active ODNs. This result is partly consistent with the conclusion drawn by Patzel *et al *[17]. In contrast to the conclusion of Ding and Lawrence [33], although *f*_*max *_is revealed to be efficacy-associated, the peak value of the target site SSPP correlates negatively with efficacy.

The helical region in the target site appears to be more important, as suggested by Laptev [20], because features *f*_*PP *_and *f*_*CP *_satisfy our selection criteria for ODN classification. From the analysis, it is obvious that the structural consistency features, *f*_*rms*_, *f*_*peak*_, and *f*_*difference*_, are more important in target site selection. But this should not be interpreted as implying simple correspondences between structural consistency and efficacy.

ODN efficacy may be closely associated with the local structures of the 5' and 3' termini of the target sites. Fisher classifiers using factors *f*_5*S *_and *f*_3*S *_gave high specificity or sensitivity in ODN discrimination.

Although some features are efficacy-associated, the relationship between structural factors and efficacy is highly complex. No single feature has been found to correlate highly with efficacy, and no feature is reliable on its own for distinguishing active from inactive ODNs. Two feature sets defined on the SSPP and SUP representations of the target site are selected as inputs of efficacy-predicting neural networks: *F*_*SSPP *_= {*f*_*rms*_, *f*_*max*_, *f*_*peak*_, *f*_*difference*_} and *F*_*SUP *_= {*f*_*PP*_, *f*_*CS*_, *f*_*CP*_, *f*_5*S*_, *f*_3*S*_}.

### Efficacy predicting using neural networks

To assess the ability of selected features to predict efficacy, two neural network models were constructed, one for features defined on the SSPP and the other for features derived from the SUP sequence representation of the target structure.

Previous studies have shown that cross-validation is important for estimating accuracy [11-14]. Since ODNs always have similar properties if they are near each other on the same gene or are measured in the same study, the network training process should be completely independent of the test data [12,13]. In this research, cross-validation was done by the "minus-one-gene" (-gene) [13] approach. ODNs targeting to 8 mRNAs (listed in Table 6) were selected alternately from the dataset for testing, while the remainder, assayed in the same studies, were used as the training set. The test mRNA selection criteria were: (a) more than 15 different target sites were tested; (b) the efficacy of at least one ODN was greater than 75%.

Sixteen neural networks for efficacy prediction were tested in our cross-validation experiments. The network group N_SSPP _(N_SSPP_1~N_SSPP_8) took *F*_*SSPP *_as inputs, and the N_SUP _group (N_SUP_1~N_SUP_8) took *F*_*SUP *_as the input parameter set. The outputs of all these networks met the condition of convergence within 100 training cycles.

Several methods have been used to measure the accuracy of ODN predictors [11-14]. To obtain rounded assessments for the aforementioned neural networks, two different types of indices were computed: (1) specificity *S*_*P*_, sensitivity *S*_*e *_and accuracy Acc=Tn+TpTp+Tn+Fp+Fn
 MathType@MTEF@5@5@+=feaafiart1ev1aaatCvAUfKttLearuWrP9MDH5MBPbIqV92AaeXatLxBI9gBaebbnrfifHhDYfgasaacH8akY=wiFfYdH8Gipec8Eeeu0xXdbba9frFj0=OqFfea0dXdd9vqai=hGuQ8kuc9pgc9s8qqaq=dirpe0xb9q8qiLsFr0=vr0=vr0dc8meaabaqaciaacaGaaeqabaqabeGadaaakeaacqWGbbqqcqWGJbWycqWGJbWycqGH9aqpdaWcaaqaaiabdsfaunaaBaaaleaacqWGUbGBaeqaaOGaey4kaSIaemivaq1aaSbaaSqaaiabdchaWbqabaaakeaacqWGubavdaWgaaWcbaGaemiCaahabeaakiabgUcaRiabdsfaunaaBaaaleaacqWGUbGBaeqaaOGaey4kaSIaemOray0aaSbaaSqaaiabdchaWbqabaGccqGHRaWkcqWGgbGrdaWgaaWcbaGaemOBa4gabeaaaaaaaa@4585@ calculated using fixed threshold values, as mentioned above in the account of feature selection; (2) the receiver operating characteristics (ROC) curve [41], which is a plot of *S*_*e *_versus 1 - *S*_*P *_at different thresholds. The ROC area was calculated as a quantitative indicator of the ability of the network to classify. The cutoff efficacy value used to distinguish positive from negative ODNs in the cross-validation test was 75%.

**Figure 3:**
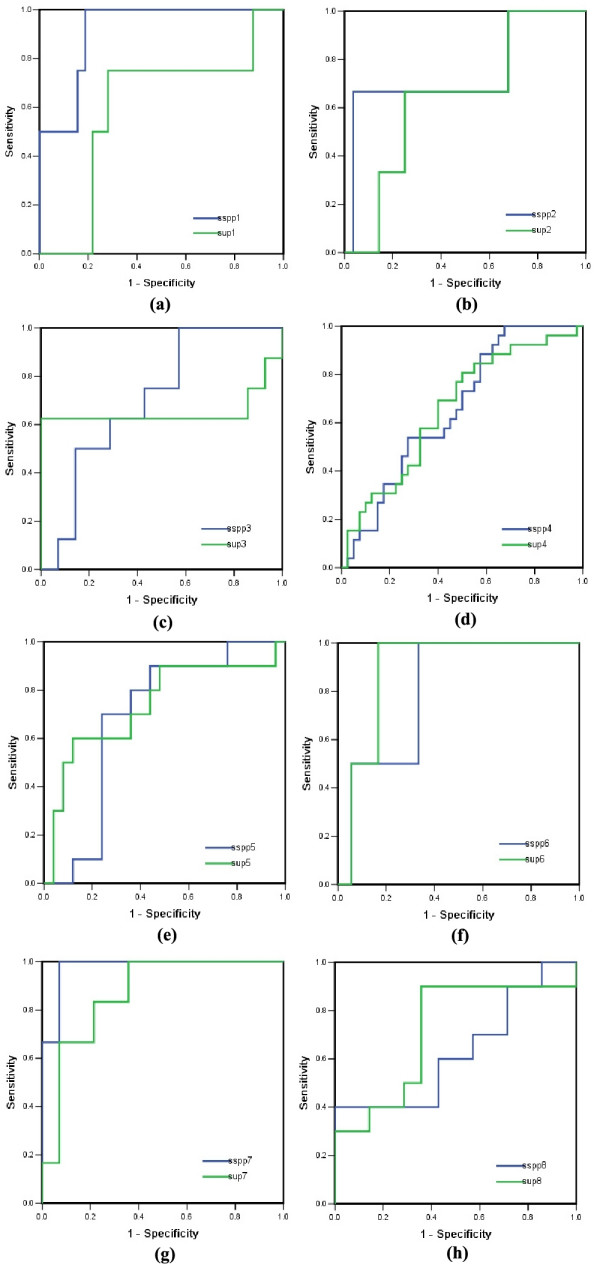
ROC curves for efficacy-predicting neural networks. ROC curves are shown for networks (a) N_SSPP_1 and N_SUP_1; (b) N_SSPP_2 and N_SUP_2; (c) N_SSPP_3 and N_SUP_3; (d) N_SSPP_4 and N_SUP_4; (e) N_SSPP_5 and N_SUP_5; (f) N_SSPP_6 and N_SUP_6; (g) N_SSPP_7 and N_SUP_7; (h) N_SSPP_8 and N_SUP_8.

The performances of the neural networks are listed in Table 7. The specificities, *S*_*P*_, of all the networks in these two groups are greater than the related sensitivities, *S*_*e*_. This performance is beneficial for ODN design, since users will only be interested in candidates with high predicted efficacy in practical applications [14]. The ROC curves of the 16 networks tested on ODNs targeting to 8 different mRNAs are shown in Figure 3. The best ROC curve areas were obtained in cross-validation experiment 7 (network N_SSPP_7 and N_SUP_7), which used the data from Matveeva *et al*. [6] as test set. The average ROC area for N_SUP _is 0.77. The average for N_SUP _is 0.73, which is little lower.

## Discussion

Compared with most other bioinformatics research problems, studies on computer-aided ODN design are far from "data rich". Moreover, the data collected from the published literature are variable owing to the diversity of experimental methods. To provide a more reliable basis for feature-mining and predictor development, one focus of future work will be on enlargement of the dataset. A large dataset with quality control will make the analysis and cross-validation of grouped homogeneous subsets possible, and therefore make the ODN design systems more reliable.

Another "data poor" limitation in our study and related research [6,17,29] is that not all possible target RNA structures are taken into account. As pointed out by Mathews, an ideal way to integrate the predicted RNA structures would be to compute a partition function, which sums the contributions of all structures weighted by their Boltzmann probabilities [44]. However, the determination of a partition function has *O*(*N*^3^) computational complexity [45], so this method is practicable only for short RNA sequences. Several studies have been done on the estimation of partition function with lower computational cost [44,46-48]. The Vienna RNA secondary structure prediction server [49] can now compute the partition function of RNA up to 5000 bases for batch jobs. One implication of this study that warrants further investigation is ODN design using the partition function of the target mRNA, which is based on more reliable structural information.

The factors influencing the potential of an ODN are complex and so far poorly understood. Although this paper focuses on the relationship between ODN efficacy and target site structure, we do not ignore other factors that have been shown to influence efficacy, such as chemical properties, DNA-RNA duplex stability, sequence motifs, metabolic properties of target mRNA, *etc*. [4]. We do believe that as more factors are considered in ODN efficacy prediction, the more reliable the target site selection becomes.

## Conclusion

This paper presents a method, based on multiple predicted target mRNA structures, for reducing the uncertainty of structure prediction in ODN design. Several efficacy-associated features characterizing the integrated structure of the target site have been discovered. The structural consistency features of the target seem to be correlated with efficacy. In contrast, some features of favorable ODN targets reported in previous research, which emphasized single-stranded regions, were found to correlate weakly with efficacy. In addition, the local structures of the 5' and 3' termini were shown to be important in target site selection.

Neural network efficacy predictors using features defined on integrated structures as inputs have been shown to perform well, implying that these features can also be used for other forms of efficacy prediction such as Bayesian statistics (BS), multiple linear regression (MLR), decision tree (DT) and support vector machine (SVM).

## Methods

After preliminary experiments, feed-forward network architecture with a hidden layer containing 20 nodes was applied to each network. The input neurons used a logarithmic sigmoid (tan-sigmoid) activation function; the output neurons used a hyperbolic tangent sigmoid (log-sigmoid) activation function. The weights and bias values of the networks were updated according to the Levenberg-Marquardt optimization algorithm [42], which appears to be the fastest method for training a moderate-size feed-forward neural network [43]. Matlab^® ^Neural Network Toolbox 4.0.3 was used for all neural network implementation.

## Authors' contributions

SW guided the project. XB and SW conceived of the study. XB wrote program, analyzed the results and drafted the manuscript. SL and DS helped in dataset construction. WS and JY helped in analysis and discussion, gave useful comments.

## Figures and Tables

**Table 1:** Summary of antisense target genes and their predicted structures used in this study

Accession	Description	No. structures	No. ODNs
X62295	Rattus mRNA for vascular type-1 angiotensin II receptor.	50	36
XM_051583	Homo sapiens v-raf-1 murine leukemia viral oncogene homolog 1 (RAF1), mRNA	50	31
M14758	Homo sapiens P-glycoprotein (PGY1) mRNA	50	22
NM_004996	Homo sapiens ATP-binding cassette, sub-family C (CFTR/MRP), member 1 (ABCC1), transcript variant 1, mRNA	50	14
M24283	Human intercellular adhesion molecule-1 (ICAM-1)	50	66
X52479	Human PKC alpha mRNA for protein kinase C alpha	37	19
NM_001078	Homo sapiens vascular cell adhesion molecule 1 (VCAM1), transcript variant 1, mRNA	50	35
XM_057446	Homo sapiens selectin E (endothelial adhesion molecule 1) (SELE), mRNA.	50	11
M30640	Human endothelial leukocyte adhesion molecule I (ELAM1) mRNA, complete cds	50	4
NM_000877	Homo sapiens interleukin 1 receptor, type I (IL1R1), mRNA.	50	20
M31585	Mouse (clone lambda-c5e) intercellular adhesion molecule 1 (ICAM-1) mRNA, complete cds	39	8
BC036531	Homo sapiens collagen, type I, alpha 1, mRNA (cDNA clone MGC:33668 IMAGE:5264710)	50	19
NM_010784	Mus musculus midkine (Mdk), mRNA	17	4
M15077	P.pyralis (firefly) luciferase gene, complete cds	39	8
X03484	Human mRNA for raf oncogene	50	20
X14805	Mus musculus mRNA for DNA methyltransferase 1	50	8
BC005976	Homo sapiens ras homolog gene family, member A, mRNA	23	13
M10843	Rabbit beta-globin mRNA	26	24
U45880	Human X-linked inhibitor of apotosis protein XIAP mRNA	36	6
AF015950	Human telomerase reverse transcriptase mRNA	50	5
NR_001566	Homo sapiens telomerase RNA component (TERC) on chromosome 3	23	5
M34309	Human epidermal growth factor receptor (HER3) mRNA, complete cds.	50	22
NM_004507	Homo sapiens HUS1 checkpoint homolog (S. pombe) (HUS1), mRNA.	33	11
AJ278710	Escherichia coli 23S rRNA gene, strain K12 DSM 30083T	50	7
X03363	Human c-erb-B-2 mRNA	50	3
M10988	Human tumor necrosis factor (TNF) mRNA	26	4
NM_000791	Homo sapiens dihydrofolate reductase (DHFR), mRNA.	50	7
NM_001168	Homo sapiens baculoviral IAP repeat-containing 5 (survivin) (BIRC5), mRNA	29	5
NM_013642	Mus musculus dual specificity phosphatase 1 (Dusp1), mRNA	37	8
AF025846	Co-reporter vector pRL-TK, complete sequence	50	4

**Table 2:** Parameters derived from the SSPP representation

Parameter	Definition
*f*_*mean*_	Mean, fmean=1n∑i=1npi MathType@MTEF@5@5@+=feaafiart1ev1aaatCvAUfKttLearuWrP9MDH5MBPbIqV92AaeXatLxBI9gBaebbnrfifHhDYfgasaacH8akY=wiFfYdH8Gipec8Eeeu0xXdbba9frFj0=OqFfea0dXdd9vqai=hGuQ8kuc9pgc9s8qqaq=dirpe0xb9q8qiLsFr0=vr0=vr0dc8meaabaqaciaacaGaaeqabaqabeGadaaakeaacqWGMbGzdaWgaaWcbaGaemyBa0MaemyzauMaemyyaeMaemOBa4gabeaakiabg2da9maalaaabaGaeGymaedabaGaemOBa4gaamaaqahabaGaemiCaa3aaSbaaSqaaiabdMgaPbqabaaabaGaemyAaKMaeyypa0JaeGymaedabaGaemOBa4ganiabggHiLdaaaa@40E5@
*f*_*rms*_	Root mean square, frms=1n∑i=1n(pi−fmean)2 MathType@MTEF@5@5@+=feaafiart1ev1aaatCvAUfKttLearuWrP9MDH5MBPbIqV92AaeXatLxBI9gBaebbnrfifHhDYfgasaacH8akY=wiFfYdH8Gipec8Eeeu0xXdbba9frFj0=OqFfea0dXdd9vqai=hGuQ8kuc9pgc9s8qqaq=dirpe0xb9q8qiLsFr0=vr0=vr0dc8meaabaqaciaacaGaaeqabaqabeGadaaakeaacqWGMbGzdaWgaaWcbaGaemOCaiNaemyBa0Maem4Camhabeaakiabg2da9maakaaabaWaaSaaaeaacqaIXaqmaeaacqWGUbGBaaWaaabCaeaadaqadaqaaiabdchaWnaaBaaaleaacqWGPbqAaeqaaOGaeyOeI0IaemOzay2aaSbaaSqaaiabd2gaTjabdwgaLjabdggaHjabd6gaUbqabaaakiaawIcacaGLPaaadaahaaWcbeqaaiabikdaYaaaaeaacqWGPbqAcqGH9aqpcqaIXaqmaeaacqWGUbGBa0GaeyyeIuoaaSqabaaaaa@4A69@
*f*_*max*_	Maximum, fmax⁡=max⁡i=1,⋯,n{pi} MathType@MTEF@5@5@+=feaafiart1ev1aaatCvAUfKttLearuWrP9MDH5MBPbIqV92AaeXatLxBI9gBaebbnrfifHhDYfgasaacH8akY=wiFfYdH8Gipec8Eeeu0xXdbba9frFj0=OqFfea0dXdd9vqai=hGuQ8kuc9pgc9s8qqaq=dirpe0xb9q8qiLsFr0=vr0=vr0dc8meaabaqaciaacaGaaeqabaqabeGadaaakeaacqWGMbGzdaWgaaWcbaGagiyBa0MaeiyyaeMaeiiEaGhabeaakiabg2da9maaxababaGagiyBa0MaeiyyaeMaeiiEaGhaleaacqWGPbqAcqGH9aqpcqaIXaqmcqGGSaalcqWIVlctcqGGSaalcqWGUbGBaeqaaOWaaiWaaeaacqWGWbaCdaWgaaWcbaGaemyAaKgabeaaaOGaay5Eaiaaw2haaaaa@455B@
*f*_*impulse*_	Impulse factor, fimpulse=fmax⁡fmean MathType@MTEF@5@5@+=feaafiart1ev1aaatCvAUfKttLearuWrP9MDH5MBPbIqV92AaeXatLxBI9gBaebbnrfifHhDYfgasaacH8akY=wiFfYdH8Gipec8Eeeu0xXdbba9frFj0=OqFfea0dXdd9vqai=hGuQ8kuc9pgc9s8qqaq=dirpe0xb9q8qiLsFr0=vr0=vr0dc8meaabaqaciaacaGaaeqabaqabeGadaaakeaacqWGMbGzdaWgaaWcbaGaemyAaKMaemyBa0MaemiCaaNaemyDauNaemiBaWMaem4CamNaemyzaugabeaakiabg2da9maalaaabaGaemOzay2aaSbaaSqaaiGbc2gaTjabcggaHjabcIha4bqabaaakeaacqWGMbGzdaWgaaWcbaGaemyBa0MaemyzauMaemyyaeMaemOBa4gabeaaaaaaaa@45A2@
*f*_*peak*_	Peak factor, fpeak=fmax⁡frms MathType@MTEF@5@5@+=feaafiart1ev1aaatCvAUfKttLearuWrP9MDH5MBPbIqV92AaeXatLxBI9gBaebbnrfifHhDYfgasaacH8akY=wiFfYdH8Gipec8Eeeu0xXdbba9frFj0=OqFfea0dXdd9vqai=hGuQ8kuc9pgc9s8qqaq=dirpe0xb9q8qiLsFr0=vr0=vr0dc8meaabaqaciaacaGaaeqabaqabeGadaaakeaacqWGMbGzdaWgaaWcbaGaemiCaaNaemyzauMaemyyaeMaem4AaSgabeaakiabg2da9maalaaabaGaemOzay2aaSbaaSqaaiGbc2gaTjabcggaHjabcIha4bqabaaakeaacqWGMbGzdaWgaaWcbaGaemOCaiNaemyBa0Maem4Camhabeaaaaaaaa@4024@
*f*_*wave*_	Wave factor, fwave=frmsfmean MathType@MTEF@5@5@+=feaafiart1ev1aaatCvAUfKttLearuWrP9MDH5MBPbIqV92AaeXatLxBI9gBaebbnrfifHhDYfgasaacH8akY=wiFfYdH8Gipec8Eeeu0xXdbba9frFj0=OqFfea0dXdd9vqai=hGuQ8kuc9pgc9s8qqaq=dirpe0xb9q8qiLsFr0=vr0=vr0dc8meaabaqaciaacaGaaeqabaqabeGadaaakeaacqWGMbGzdaWgaaWcbaGaem4DaCNaemyyaeMaemODayNaemyzaugabeaakiabg2da9maalaaabaGaemOzay2aaSbaaSqaaiabdkhaYjabd2gaTjabdohaZbqabaaakeaacqWGMbGzdaWgaaWcbaGaemyBa0MaemyzauMaemyyaeMaemOBa4gabeaaaaaaaa@4188@
*f*_*difference*_	Mean of difference, fdifference=1n−1∑i=1n−1|pi−pi+1| MathType@MTEF@5@5@+=feaafiart1ev1aaatCvAUfKttLearuWrP9MDH5MBPbIqV92AaeXatLxBI9gBaebbnrfifHhDYfgasaacH8akY=wiFfYdH8Gipec8Eeeu0xXdbba9frFj0=OqFfea0dXdd9vqai=hGuQ8kuc9pgc9s8qqaq=dirpe0xb9q8qiLsFr0=vr0=vr0dc8meaabaqaciaacaGaaeqabaqabeGadaaakeaacqWGMbGzdaWgaaWcbaGaemizaqMaemyAaKMaemOzayMaemOzayMaemyzauMaemOCaiNaemyzauMaemOBa4Maem4yamMaemyzaugabeaakiabg2da9maalaaabaGaeGymaedabaGaemOBa4MaeyOeI0IaeGymaedaamaaqahabaWaaqWaaeaacqWGWbaCdaWgaaWcbaGaemyAaKgabeaakiabgkHiTiabdchaWnaaBaaaleaacqWGPbqAcqGHRaWkcqaIXaqmaeqaaaGccaGLhWUaayjcSdaaleaacqWGPbqAcqGH9aqpcqaIXaqmaeaacqWGUbGBcqGHsislcqaIXaqma0GaeyyeIuoaaaa@5599@

**Table 3:** Parameters derived from the SUP sequence representation

Parameter	Definition
*f*_*NS*_	Number of bases in single-stranded region
*f*_*NP*_	Number of bases in double-stranded region
*f*_*PS*_	Percentage of bases in single-stranded region to the length of ODN
*f*_*PP*_	Percentage of bases in double-stranded region to the length of ODN
*f*_*CS*_	Maximum length of consecutive subsequence in single-stranded region
*f*_*CP*_	Maximum length of consecutive subsequence in base pairing
*f*_5*S*_	Maximum length of consecutive subsequence in single-stranded region counting from 5' terminal
*f*_5*P*_	Maximum length of consecutive subsequence in base pairing counting from 5' terminal
*f*_3*S*_	Maximum length of consecutive subsequence in single-stranded region counting from 3' terminal
*f*_3*P*_	Maximum length of consecutive subsequence in base pairing counting from 3' terminal
*f*_*SC*_	Structure consistency, fSC=1n−1∑i=1n−1E(Si,Si−1) MathType@MTEF@5@5@+=feaafiart1ev1aaatCvAUfKttLearuWrP9MDH5MBPbIqV92AaeXatLxBI9gBaebbnrfifHhDYfgasaacH8akY=wiFfYdH8Gipec8Eeeu0xXdbba9frFj0=OqFfea0dXdd9vqai=hGuQ8kuc9pgc9s8qqaq=dirpe0xb9q8qiLsFr0=vr0=vr0dc8meaabaqaciaacaGaaeqabaqabeGadaaakeaacqWGMbGzdaWgaaWcbaGaem4uamLaem4qameabeaakiabg2da9maalaaabaGaeGymaedabaGaemOBa4MaeyOeI0IaeGymaedaamaaqahabaGaemyrauKaeiikaGIaem4uam1aaSbaaSqaaiabdMgaPbqabaGccqGGSaalcqWGtbWudaWgaaWcbaGaemyAaKMaeyOeI0IaeGymaedabeaakiabcMcaPaWcbaGaemyAaKMaeyypa0JaeGymaedabaGaemOBa4MaeyOeI0IaeGymaedaniabggHiLdaaaa@4994@, where E(x,y)={1,x=y≠'U'0,x='U', or y='U'-1x≠y,x≠'S',y≠'P' MathType@MTEF@5@5@+=feaafiart1ev1aaatCvAUfKttLearuWrP9MDH5MBPbIqV92AaeXatLxBI9gBaebbnrfifHhDYfgasaacH8akY=wiFfYdH8Gipec8Eeeu0xXdbba9frFj0=OqFfea0dXdd9vqai=hGuQ8kuc9pgc9s8qqaq=dirpe0xb9q8qiLsFr0=vr0=vr0dc8meaabaqaciaacaGaaeqabaqabeGadaaakeaacqWGfbqrcqGGOaakcqWG4baEcqGGSaalcqWG5bqEcqGGPaqkcqGH9aqpdaGabaqaauaabaqadiaaaeaacqaIXaqmcqGGSaalaeaacqWG4baEcqGH9aqpcqWG5bqEcqGHGjsUcqGGNaWjcqqGvbqvcqGGNaWjaeaacqaIWaamcqGGSaalaeaacqWG4baEcqGH9aqpcqGGNaWjcqqGvbqvcqGGNaWjcqGGSaalcqqGGaaicqWGVbWBcqWGYbGCcqqGGaaicqWG5bqEcqGH9aqpcqGGNaWjcqqGvbqvcqGGNaWjaeaacqqGTaqlcqqGXaqmaeaacqWG4baEcqGHGjsUcqWG5bqEcqGGSaalcqWG4baEcqGHGjsUcqGGNaWjcqqGtbWucqGGNaWjcqGGSaalcqWG5bqEcqGHGjsUcqGGNaWjcqqGqbaucqGGNaWjaaaacaGL7baaaaa@6622@

**Table 4:** Correlations between features and efficacy

Parameter	Pearson Correlation	Spearman Correlation	Kendall Correlation
*f*_*mean*_	-0.086	-0.055	-0.087
*f*_*rms*_	-0.150**	-0.100**	-0.147**
*f*_*max*_	-0.099*	-0.113**	-0.155**
*f*_*impulse*_	0.040	0.039	0.060
*f*_*peak*_	0.124**	0.083**	0.125**
*f*_*wave*_	-0.030	-0.017	-0.025
*f*_*difference*_	-0.094*	-0.034	-0.051
*f*_*NS*_	-0.087	-0.057	-0.082
*f*_*NP*_	-0.045	-0.043	-0.061
*f*_*PS*_	-0.073	-0.050	-0.075
*f*_*PP*_	-0.040	-0.040	-0.057
*f*_*CS*_	-0.062	-0.037	-0.053
*f*_*CP*_	-0.012	-0.012	-0.019
*f*_5*S*_	0.031	0.012	0.016
*f*_5*P*_	-0.009	-0.039	-0.055
*f*_3*S*_	-0.050	-0.011	-0.016
*f*_3*P*_	-0.036	-0.030	-0.039
*f*_*SC*_	-0.064	-0.045	-0.066

**. Correlation is significant at the 0.01 level

*. Correlation is significant at the 0.05 level

**Table 5:** Performance of Fisher linear discriminators for each parameter

Parameter	Threshold = 50%		Threshold = 75%	
		
	*S*_*e*_	*S*_*p*_	*S*_*e*_	*S*_*p*_
*f*_*mean*_	0.56	0.53	0.65	0.51
*f*_*rms*_	0.58	0.54	0.50	0.54
*f*_*max*_	0.33	0.73	0.60	0.72
*f*_*impulse*_	0.37	0.67	0.48	0.38
*f*_*peak*_	0.50	0.61	0.56	0.59
*f*_*wave*_	0.42	0.58	0.63	0.43
*f*_*difference*_	0.52	0.50	0.52	0.50
*f*_*NS*_	0.56	0.50	0.56	0.48
*f*_*NP*_	0.44	0.51	0.52	0.49
*f*_*PS*_	0.54	0.52	0.58	0.51
*f*_*PP*_	0.49	0.45	0.70	0.54
*f*_*CS*_	0.56	0.47	0.73	0.45
*f*_*CP*_	0.43	0.63	0.74	0.40
*f*_5*S*_	0.31	0.71	0.73	0.30
*f*_5*P*_	0.33	0.63	0.58	0.37
*f*_3*S*_	0.29	0.73	0.65	0.29
*f*_3*P*_	0.34	0.64	0.50	0.37
*f*_*SC*_	0.50	0.50	0.60	0.52

. high specificity (≥ 0.7) or high sensitivity (≥ 0.7)

**Table 6:** Dataset for cross-validation experiments

Networks	Accession of test gene	Number in train set	Number in test set
N_SSPP_1 and N_SUP_1	X62295	412	36
N_SSPP_2 and N_SUP_2	XM_051583	417	31
N_SSPP_3 and N_SUP_3	M14758	426	22
N_SSPP_4 and N_SUP_4	M24283	356	66
N_SSPP_5 and N_SUP_5	NM_001078	379	35
N_SSPP_6 and N_SUP_6	NM_000877	428	20
N_SSPP_7 and N_SUP_7	X03484	428	20
N_SSPP_8 and N_SUP_8	M10843	424	24

**Table 7:** The performances of two groups of networks in cross-validation experiments

Networks	Se	Sp	Acc	ROC area	Networks	Se	Sp	Acc	ROC area
N_sspp_1	0.50	0.97	0.92	0.91	N_sup_1	0	0.94	0.83	0.60
N_sspp_2	0.33	0.96	0.90	0.75	N_sup_2	0	0.93	0.84	0.69
N_sspp_3	0	0.93	0.59	0.71	N_sup_3	0	1	0.64	0.65
N_sspp_4	0	1	0.88	0.66	N_sup_4	0.5	0.86	0.82	0.66
N_sspp_5	0	1	0.71	0.69	N_sup_5	0	1	0.71	0.74
N_sspp_6	0	0.94	0.85	0.81	N_sup_6	0	1	0.9	0.89
N_sspp_7	0	1	0.70	0.98	N_sup_7	0.17	0.93	0.70	0.89
N_sspp_8	0	1	0.58	0.63	N_sup_8	0.40	0.86	0.67	0.71

. high specificity (≥ 0.7) or high sensitivity (≥ 0.7)
